# Differential gene expression profiles are dependent upon method of peripheral blood collection and RNA isolation

**DOI:** 10.1186/1471-2164-9-474

**Published:** 2008-10-10

**Authors:** Adam L Asare, Svetlana A Kolchinsky, Zhong Gao, Richard Wang, Khadir Raddassi, Katarzyna Bourcier, Vicki Seyfert-Margolis

**Affiliations:** 1 University of California, San Francisco, Immune Tolerance Network, 3 Bethesda Metro Suite 400, Bethesda, MD 20814, USA

## Abstract

**Background:**

RNA isolation and purification steps greatly influence the results of gene expression profiling. There are two commercially available products for whole blood RNA collection, PAXgene™ and Tempus™ blood collection tubes, and each comes with their own RNA purification method. In both systems the blood is immediately lysed when collected into the tube and RNA stabilized using proprietary reagents. Both systems enable minimal blood handling procedures thus minimizing the risk of inducing changes in gene expression through blood handling or processing. Because the RNA purification steps could influence the total RNA pool, we examined the impact of RNA isolation, using the PAXgene™ or Tempus™ method, on gene expression profiles.

**Results:**

Using microarrays as readout of RNA from stimulated whole blood we found a common set of expressed transcripts in RNA samples from either PAXgene™ or Tempus™. However, we also found several to be uniquely expressed depending on the type of collection tube, suggesting that RNA purification methods impact results of differential gene expression profiling. Specifically, transcripts for several known PHA-inducible genes, including IFNγ, IL13, IL2, IL3, and IL4 were found to be upregulated in stimulated vs. control samples when RNA was isolated using the ABI Tempus™ method, but not using the PAXgene™ method (p < 0.01, FDR corrected). Sequenom Quantiative Gene Expression (QGE) (SanDiego, CA) measures confirmed IL2, IL4 and IFNγ up-regulation in Tempus™ purified RNA from PHA stimulated cells while only IL2 was up-regulated using PAXgene™ purified (p < 0.05).

**Conclusion:**

Here, we demonstrate that peripheral blood RNA isolation methods can critically impact differential expression results, particularly in the clinical setting where fold-change differences are typically small and there is inherent variability within biological cohorts. A modified method based upon the Tempus™ system was found to provide high yield, good post-hybridization array quality, low variability in expression measures and was shown to produce differential expression results consistent with the predicted immunologic effects of PHA stimulation.

## Background

Microarrays have rapidly become the assay of choice for clinical investigators wanting to measure gene expression, owing to their high-throughput and relative ease of use. As with any assay, it is critical that experimental variance is minimized in order to permit measurement of true biological variance. In clinical microarray studies, the sources of experimental variance can be considerable. While a range of corrections exist to detect and correct for variability introduced during hybridization and due to chip quality, little attention has been paid to the impact of specimen collection, handling and processing on the resulting gene expression measures.

For immunological studies, peripheral blood is commonly used in microarray experiments, as it is the most easily obtained source of lymphocytes, granulocytes, and other cells that may provide insight into immune function. Historically, gradient density-based methods have been used to purify white blood cells from peripheral blood. However, it is known that, within minutes of collection, peripheral blood gene expression profiles change significantly due to transcript induction and transcript degradation[1], and it is almost certain that the purification process introduces further changes in expressed transcripts.

To address these concerns, RNA whole blood collection tubes have been developed that have the considerable advantage of lysing whole blood at the time of collection, while simultaneously stabilizing RNA for later purification. The PAXgene™ Whole Blood RNA isolation system contains a proprietary solution that reduces RNA degradation and transcript induction upon peripheral blood collection [2,3]. Using the Qiagen total RNA isolation method, this system has been evaluated for clinical applications using RT-PCR[4] and Affymetrix microarrays [3,5]; in some cases it has shown a lack of concordance in gene expression with other isolation methods[2,6,7]. The Tempus™ Whole Blood RNA isolation system offers an alternative approach to peripheral blood RNA isolation, again using a proprietary solution to directly lyse whole blood and stabilize RNA. There have, as yet, been no reports of the efficiency or accuracy of the Tempus™ Whole Blood RNA isolation system or comparisons to other methods.

In this report, we compare these two whole blood RNA purification methods for use in microarray experiments to measure immune response gene expression, and show that the choice of RNA purification method can have significant implications. Using phytohemagglutinin (PHA) stimulated whole blood as a test case, we found that RNA yield and hybridization quality indicators were better for RNA isolated using the Tempus™ RNA purification method. While PHA induced a set of transcript expression changes detected in both Tempus™ and PAXgene™ samples, use of the Tempus system resulted in the identification of a greater number of gene expression changes that would be expected to result from PHA stimulation.

## Results

### RNA yield and hybridization quality indicators

**Figure 1:**
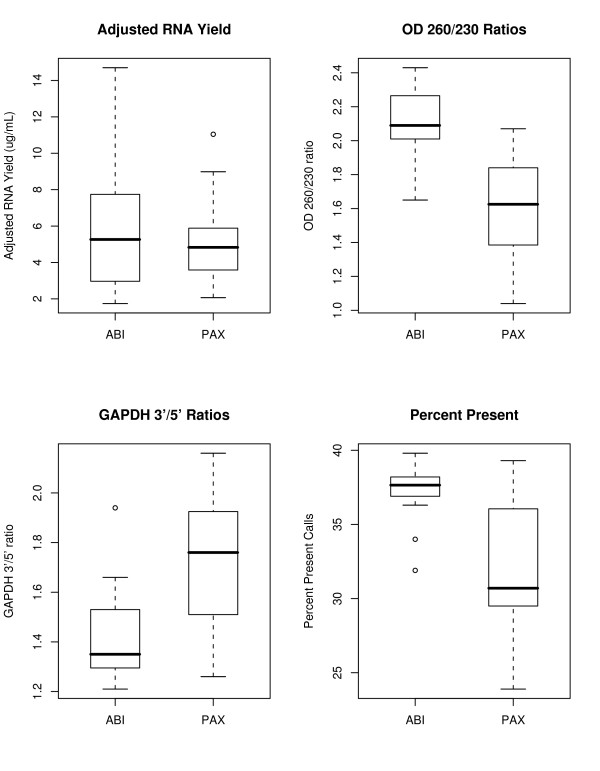
**Differences in RNA quality and yield between PAXgene™ and Tempus™.** The tempus system has higher mean yields, improved RNA purity based on OD 260/230 ratios, less degradation based on GAPDH 3'/5 ratios, and a higher number of expressed transcripts based on Percent Present Calls.

RNA yield, RNA purity, and post-hybridization quality indicators were compared for specimens collected directly into the two whole blood RNA isolation system tubes. Slightly higher mean RNA yields were observed in samples isolated using the Tempus™ system compared to PAXgene™ (5.6 μg/ml and 5.01 μg/ml, respectively, Fig. 1), with Tempus™ samples showing greater variability. RNA yield was adjusted for blood collection starting volumes, which was 3 ml for Tempus ™ and 2.5 ml for PAXgene™. Tempus™-collected specimens yielded a higher purity of RNA, based on higher OD_260/230_ratios (Fig. 1) and higher "Percent Present Calls", an indicator of the number of transcripts reliably detected (Fig. 1). In addition, RNA isolated by the Tempus™ methodology showed decreased GAPDH 3'/5' ratios. This is an indicator for RNA degradation where cRNA synthesis is 3' biased, hence the more degraded the RNA the less signal for probes detecting the 5' end of transcripts (Fig. 1).

### Controlling for Li Heparin

We aimed to compare gene expression profiles of peripheral blood RNA from cells stimulated by phytohemagglutinin (PHA), a mitogen known to induce expression of immune activation transcripts including those for IL-2, IL-4, and IFNγ[8]. However, because blood collection with either Tempus™ or PAXgene™ systems results in immediate lysis of peripheral blood cells, blood had to be collected first and subsequently PHA-stimulated in Li Heparin tubes (Becton-Dickenson, San Jose, California), prior to transfer to the test systems.

To control for the effect of Li Heparin tube collection, we performed an initial comparison of 5 healthy control samples drawn directly in PAXgene™ or Tempus™ tubes vs. the same 5 healthy control samples drawn in Li Heparin tubes with no PHA stimulation. RNAs were hybridized to the HG-U133 2.0 Plus Affymetrix GeneChip^® ^microarray. Only two transcripts, for the B-cell translocation transcript BTG-1, were identified as down-regulated in samples collected with Li Heparin tubes, compared to those collected in Tempus tubes. Interestingly, just one transcript was up-regulated using PAXgene™ tubes, corresponding to one of the transcripts up-regulated in the Tempus™ system, BTG1. These results suggest that collection of samples into Li Heparin tubes prior to PHA stimulation had a minimal effect on differential expression and would have negligible effect on the interpretation of differences between the two tube types.

### Detection of transcriptional response to PHA stimulation

To compare PAXgene™ and Tempus™ tubes, whole blood samples collected in Li Heparin tubes from 7 healthy controls were split into PHA-stimulated or unstimulated aliquots. After 3 hrs, samples were transferred to each respective RNA isolation system and hybridized to the HG-U133 2.0 Plus Affymetrix GeneChip^® ^microarray.

**Figure 2:**
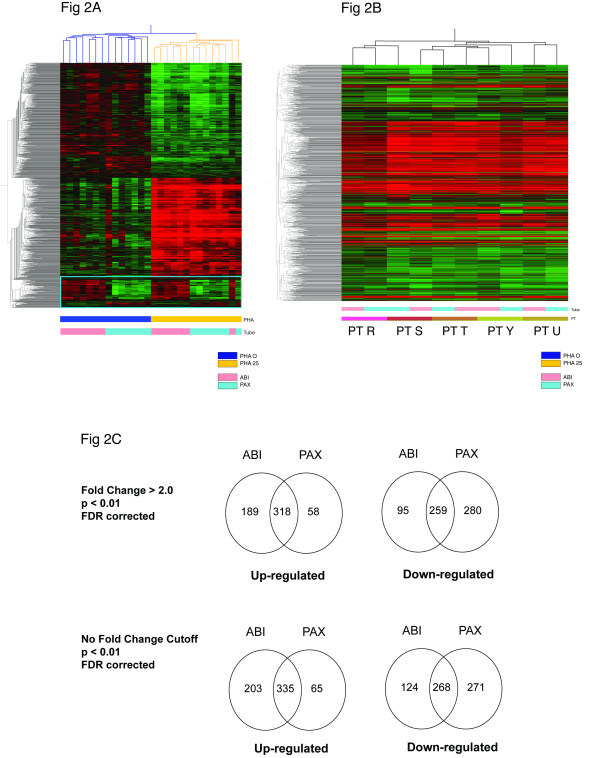
**A Hierarchical clustering of individual samples for the 1,338 transcripts generated from a union set of transcripts statistically significant as either up- or down-regulated using Tempus™ or PAXgene™ tubes.** PHA stimulation is associated with the primary segregation of samples (Blue and Gold Bar), with RNA collection tube as a secondary grouping (Pink and Teal). **B**. Hierarchical clustering of fold change values within a participant/tube condition for PHA stimulation vs. no PHA stimulation shows consistent grouping by participant despite difference in collection tubes (Note color bars by participant). **C**. Venn diagram for comparisons of 3 hr PHA stimulation vs. 3 hrs with no stimulation. Substantial up-regulated transcripts using Tempus™ compared to PAXgene™, and a greater number of down-regulated transcripts using PAXgene™ compared to Tempus™.

Hierarchical clustering of absolute expression levels, irrespective of gene function, showed that the primary separation occurred between stimulated vs. unstimulated samples (as opposed to different sample collection systems). The clustering was generated on the 28 individual samples using the 1,266 differentially expressed transcripts that were either up- or down-regulated by PHA-stimulation (Fig. 2A). Stimulation condition forms a primary division, followed by a secondary split based on PAXgene™ or Tempus™ collection. However, hierarchical clustering of the change in expression level between nonstimulated and stimulated samples per participant showed a fair degree of consistency across PAXgene™ and Tempus™ systems, as illustrated in Figure 2B. In order to determine how use of either platform influences measures of gene expression of the transcripts of interest, a breakdown of overlap and concordance of up-regulated and down-regulated transcripts for each platform was performed.

**Figure 3:**
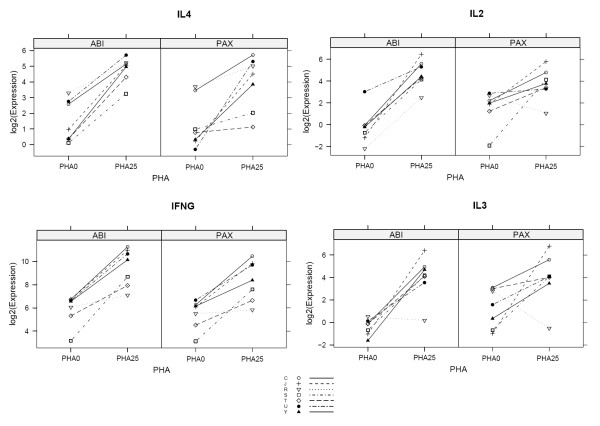
**Differential expression of individual subjects for four transcripts known to be differentially expressed upon addition of PHA: IL2, IL4, IFNG, and IL3.** Using the Tempus ABI system, all four transcripts are identified as up-regulated by PHA stimulation, with an FDR-adjusted p value < 0.01 and a fold change > 2. These four transcripts are not identified using the same criteria from data generated using PAX system.

PHA-stimulation resulted in 538 transcripts being detected as upregulated, and 392 downregulated when the Tempus™ system was used. For the PAXgene™ system, 400 were found to be upregulated and 539 downregulated (p < 0.01, FDR) by PHA stimulation (Fig. 2C). Among all of these, 335 upregulated (56% of the union set) and 268 downregulated transcripts (40% of the union set) were common between the two platforms. Among the 203 upregulated immune function transcripts uniquely identified by the Tempus™ Whole Blood RNA isolation system were IL-2, IL-3, IL-4, IFNγ, STAT3, IRF4, IL-21R, CLA4, CD44, BCL6 and STAT1. In contrast, the 65 upregulated immune function transcripts uniquely identified by the PAXgene™ Whole Blood RNA isolation system included IL-2RA, BCL6 (alternate transcript), IL-1RN, CCL18, and CD48 (Additional file 1). Expression profiles of several transcripts known to be up-regulated by PHA such as IL-2, IL-3, IL-4, and IFNγ [9] are illustrated for all 7 subjects in Fig 3A. Samples isolated using PAXgene™ preparation resulted in 271 transcripts uniquely identified as down-regulated, while Tempus™ tube preparation resulted in 124 transcripts not identified as down-regulated by PAXgene™ (Fig. 2A, B, C) (Additional file 2).

**Figure 4:**
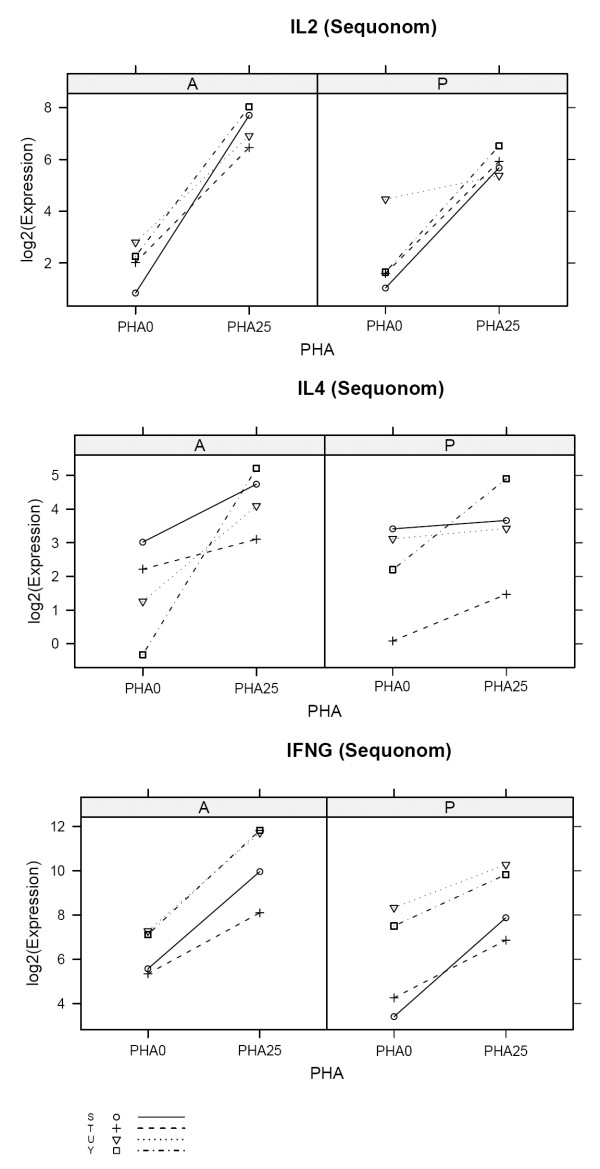
**Sequenom QGE assay: Tempus™ appears to have slightly higher differential expression estimates than PAXgene™ for IL2, IL4 and IFNG.** IL4, IFNγ, were statistically significant for Tempus ™ samples (P < 0.05). Both Tempus™ and PAXgene™ systems show IL2 as up-regulated (p < 0.05).

To validate differential expression measured by microarray, real time PCR was performed on four of the seven samples that had sufficient RNA remaining. Using the Sequenom Quantitative Gene Expression (QGE) platform, eighteen immune function transcripts were assessed, including IL-2, IL-4, IFNγ, controls and others not known to be regulated by PHA. Other transcripts assessed include: TGFβ, P19, Perforin, MIG, IP10, IL10, GB, FOXP3, CXCR3, CTLA4, CTGF, CD3, CD25, CD20, and CD103. Transcripts for IFNγ, IL-2, IL-4 were statistically significant for differential expression between un-stimulated and stimulated samples using the Tempus™ system (p < 0.05). Samples prepared using the PAXgene™ system exhibited greater variability in transcript levels in both unstimulated and stimulated conditions, thus trending towards upregulation for IL-2, IL-4, and IFNγ, but not statistically significant (fig 4).

## Discussion

While there are an increasing number of studies using microarrays and/or quantitative real time PCR as immune monitoring tools in clinical trials, there have been few studies assessing the contribution of the RNA purification method to the expressed gene profile. Here, we have demonstrated that the choice of RNA preparation method can have significant influence on resulting gene expression measures.

Using PHA-stimulated versus unstimulated whole blood as a model, we anticipated that a large number of transcripts would be differentially expressed, given that PHA is global activator of human peripheral T lymphocytes and has been shown to induce a number of immune response transcripts, including several cytokines. Indeed, microarray analysis revealed that PHA altered the expression of a large number of transcripts, regardless of whether RNA purification was performed by Tempus™ or PAXgene™ methods. While both methods revealed a common set of 376 transcripts whose expression was altered by PHA-stimulation, Tempus™-prepared specimens revealed an additional 189 up-regulated transcripts that were not detected in PAXgene™-prepared specimens, many of which fall into the category of immune response or cellular proliferation transcripts. Notable in this group are immune response transcripts such as the cytokines IL-2, IL-4 and IFNγ, which are known to be induced by PHA[7]. Transcriptional changes could not be attributed to differences in cell numbers since 3 hours of stimulation by PHA is not long enough to increase cell numbers.

Such differences in expression results may reflect differences in quality of the purified RNA: in these experiments, use of the PAXgene™ resulted in poorer RNA quality and post-hybridization metrics compared to specimens prepared using the Tempus system. In addition, RNA yields, on average, were higher using the Tempus™ system, as were microarray quality assurance measures such as RNA Purity and Percent Present Calls.

In human gene expression profile studies, the combination of biological variation as well as technical variation from sample to sample plays a greater role than in many other experimental settings. This is primarily due to the fact that there is no ability to repeat a condition and that it is not possible to provide technical replicates for each condition, meaning that each individual patient for a given condition or treatment is treated as a biological "replicate". In this case, the differential expression between unstimulated and PHA-stimulated transcripts with respect to fold-change across patients was more variable with PAXgene™ RNA. Mean expression estimates for immune function transcripts most often had higher standard errors that led to higher p-values using the PAXgene™ system.

## Conclusion

Overall these findings have important implications for the use of gene expression profiling to monitor immune system changes in humans. Given the biological variation of humans and the differences in sample handling inherent in multicenter clinical trials, controlled sample processing and quality is essential to ensure valid results. There may be advantages and disadvantages to each platform based on issues such as blood volume, cost, or need for automation. These studies demonstrate that the choice of platform, and its associated methods, is important for defining expressed transcripts, and that reproducibility and quality in RNA preparation are critical to define changes in expressed gene profiles that meet the statistical rigor necessary for interpretation and validation of signatures for clinical trial monitoring.

## Methods

### Study Design and Blood Collection

Our objective was to assess the effect of two available blood collection systems with regard to differential gene expression in clinical samples. The primary comparison was of unstimulated peripheral blood samples with those stimulated *ex vivo *with phytohemagglutinin (PHA) (Remel, Kansas, P/N HA16/30852801). Both PAXgene™ and Tempus™ whole blood collection tubes were used in this step. Since cells are immediately lysed upon collection with either PAXgene™ or Tempus™, blood had to be initially drawn in Li Heparin tubes (P/N 367880, BD, Franklin Lakes, NJ) prior to transfer into either system. A secondary comparison was made between samples drawn directly in both collection systems versus. Samples were drawn in Li Heparin and then immediately transferred into their respective tube types, to determine if Li Heparin adversely effected gene expression on its own.

For these comparisons, whole blood was collected from seven healthy individuals who provided informed consent, under the approval of the Institutional Review Board of Brigham and Women's Hospital. A total of 110 mL of peripheral blood was collected from each participant using Li Heparin tubes. Whole blood from individual participants was pooled into a 200 mL plastic container and a set of seven aliquots from the pool was incubated for 3 hrs at room temperature with no stimulant added; a second set of seven aliquots was stimulated with 25 μg/mL of PHA and incubated for 3 hrs. After incubation, samples were subsequently transferred to either PAXgene™ or Tempus™ tubes. To assess whether stimulation by PHA was successful, FACS detection for IFNγ (BD, Franklin Lakes, NJ) was performed. Samples from five of the seven subjects were drawn directly into PAXgene™ and Tempus™ tubes for assessing the effect of Li Heparin alone as described above. In total, 48 samples were collected and hybridized as part of the analysis.

### RNA Isolation and quality assessment

RNA was extracted at the ITN Central Nucleic Acid Isolation Core Facility in Pittsburgh, PA according to the ITN-modified method for the Tempus™ system. The PAXgene™ Whole Blood RNA samples were processed using the PAXgene™ Blood RNA Kit based on the Qiagen method for column purification of nucleic acids (Part Number 762134, Qiagen). Whole blood samples collected into Tempus™ vacuette were extracted using ABI Prism™ 6100 Nucleic Acid PrepStation™ and using Tempus™ extraction reagents. Samples were frozen immediately at -70°C upon collection. Extraction steps included addition of PBS buffer to compensate for short blood sample draws, a wash step with Purification Wash Solution 1 two times at 80% vacuum for 500 seconds, followed by a single washing step using Purification Wash Solution 2 at 80% vacuum for 120 seconds. An extra vacuum step was performed to eliminate Purification Wash Solution 2 from the filter. Upon changing the reservoirs on the ABI 6100 PrepStation™, three more washing steps were performed using Purification Wash Solution 2 at 80% vacuum for 120 seconds. At the elution step, adapter plates were changed one extra time to ensure the purity of the eluted RNA. Eluted RNA was then concentrated using the Microcon YM-100 (Millipore, Billerica, MA). RNA purity and yield were assessed prior to hybridization using the Agilent 2100 Bioanalyzer (Agilent Technologies, Palo Alto, CA) [10-12]

### Processing of Microarrays and *MassARRAY Quantitative Gene Expression (QGE)*

The ITN Central Microarray Facility (Expression Analysis, Durham, NC) performed all hybridizations with the Affymetrix HG-U133 2.0 Plus microarray. All processing was done according to manufacturer's instructions. Globin reduction was performed followed by cRNA target amplification using the Affymetrix In-vitro Transcription (IVT) Kit. Standard pre- and post-hybridization quality control metrics were used to assess sample processing and hybridization quality. Microarrays have been deposited within GEO (Accession number GSE12711).

### Confirmation by MassARRAY Quantitative Gene Expression (QGE) Analysis

Multiplexed primer and competitive template designs were created using the MassARRAY QGE Assay Design software v1.0 (Sequenom, San Diego, CA) for random hexamer priming, such that at least one PCR primer spanned an exonic boundary per each transcript assayed. The 20 gene panel assayed for this study was designed as a single 20-plex reaction.

Copy number determination for each transcript was conducted using real-time competitive PCR coupled with product resolution via Matrix-Assisted Laser Desorption/Ionization Mass Spectrometry (MassARRAY QGE, Sequenom, San Diego, CA.), as previously described [13]. Products were resolved on a linear MALDI-TOF mass spectrometer (MassARRAY Compact, Sequenom, San Diego, CA.). Signal acquisition, allele assignment and peak area integration per spectrum were conducted with the MassARRAY RT Workstation v3.4 (Sequenom, San Diego, CA). Data was analyzed using MassARRAY QGE Analyzer v3.4 (Sequenom, San Diego, CA.) with copy numbers for each transcript per sample determined based on the EC50 of standard curve titrations of known competitor amounts per assay vs. a fixed amount of cDNA template.

Normalization of copy numbers between samples for the different assays was conducted using a multiplexed set of ten well-characterized human housekeeping (normalization) transcripts plus an 18s RNA assay and geNorm software. Normalization factors per sample were calculated using the geometric mean of the most stable combination of these normalization assays, determined by the measure of their pairwise variation as calculated by geNorm[14].

### Statistical Analysis

Microarray normalization and preprocessing was performed as follows: Log-transformed Affymetrix microarray intensity values were processed using the 'threestep' function in the R/Bioconductor affyPLM package[14]; background correction was accomplished using the MASIM method (MAS5 location-dependent background correction followed by subtraction of ideal mismatch from perfect match); normalization was performed using the scaling method, to adjust the range of expression intensities across arrays; and summarization used the Tukey Biweight method to estimate a robust mean of multiple probe intensities.

The normalized data from the experiments were fit into linear models using the Bioconductor package limma[15]. The primary analysis comparing differential expression was a 2 × 2 factorial design with tube type (Tempus™, PAXgene™) and PHA stimulation (stimulation, no stimulation) as factors. The analysis to assess the effect of Li Heparin tube collection was a 2 × 2 factorial design using two factors: tube type (Tempus™, PAXgene™) and initial blood collection method (direct blood draw into Tempus™ or PAXgene™ tubes, collection in Li heparin).

Pair-wise comparisons of interest were performed using moderated t-statistics to test for significant differential expression. The Benjamini-Hochberg multiple comparison adjustment[16] was used to control false discovery rate (FDR) at the .01 level. Transcripts meeting this statistical threshold and showing a fold change > 2 were considered differentially expressed. Hierarchical clustering was performed using Agilent GeneSpring GX.

Sequenom QGE analysis was performed using the Mann-Whitney test to identify differentially expressed transcripts between samples with and without PHA stimulation using Tempus™ and PAX platforms respectively.

## Authors' contributions

ALA contributed to the study design, data analysis and drafting of the manuscript. SAK contributed to the study design, management of samples, and drafting the manuscript. ZG and RW performed the gene expression analysis. KR processed the samples for PHA stimulation and performed flow cytometry analysis. KB and VS contributed to the study design, interpreting the results in the context of immune profiling, and drafted and reviewed the manuscript.

## Supplementary Material

Additional file 1 **Transcripts up-regulated by PHA using Tempus ABI and PAXgene™ collection systems**.
Column headings are:
**Probe Affymetrix ID**
ABI 0 = Failed statistical criteria
1 = Passed statistical criteria
PAX 0 = Failed statistical criteria
1 = Passed statistical criteria
ABI-log2ratio log 2 ratios for Tempus ABI tube
ABI-p value p-value for Tempus ABI tube, FDR corrected
PAX-log2ratio log 2 ratios for PAXgene tube tube
PAX-p value p-value for PAXgene tube, FDR corrected
Symbol Gene symbol
Description Gene description
UniGene UniGene ID
OMIM OMIM ID
Pathway Pathways associated Click here for file

Additional file 2 **Transcripts down-regulated by PHA using Tempus ABI and PASGene collection systems**
Column headings are:
**Probe Affymetrix ID**
ABI 0 = Failed statistical criteria
1 = Passed statistical criteria
PAX 0 = Failed statistical criteria
1 = Passed statistical criteria
ABI-log2ratio log 2 ratios for Tempus ABI tube
ABI-p value p-value for Tempus ABI tube, FDR corrected
PAX-log2ratio log 2 ratios for PAXgene tube tube
PAX-p value p-value for PAXgene tube, FDR corrected
Symbol Gene symbol
Description Gene description
UniGene UniGene ID
OMIM OMIM ID
Pathway Pathways associated Click here for file
